# Stress and Quality of Life in Caregivers of Patients with Multiple Sclerosis

**DOI:** 10.3390/medicina60071033

**Published:** 2024-06-24

**Authors:** Anastasios Kantanis, Maria Pelantaki, Maria Lidia Fountaki, Vasilios Konstantopoulos, Themistoklis Paraskevas, Evgenia Stavropoulou, Eirini Tsiamaki, Nikolaos Trimmis, Panagiotis Plotas

**Affiliations:** 1 Laboratory Primary Health Care, School of Health Rehabilitation Sciences, University of Patras, 26504 Patras, Greece; up1047795@upnet.gr (A.K.); mariapelantaki@gmail.com (M.P.); marilidafoudaki@gmail.com (M.L.F.); konstantopoulosbill9@gmail.com (V.K.); themispara@hotmail.com (T.P.); eugenia.stavrop@gmail.com (E.S.); nicktrimmis@upatras.gr (N.T.); 2 Department of Speech and Language Therapy, School of Health Rehabilitation Sciences, University of Patras, 26504 Patras, Greece; 3 Department of Neurology, Medical School, University of Patras, 26504 Patras, Greece; eirinitsiamaki@gmail.com

**Keywords:** multiple sclerosis, caregivers, quality of life, anxiety

## Abstract

*Background and Objectives*: Multiple sclerosis is a chronic demyelinating disease of the central nervous system. Individuals with multiple sclerosis may require daily care and support from caregivers due to the disease’s progressive and unpredictable nature. However, the role of caregiving is not without its challenges, and caregivers themselves often face significant repercussions in terms of their quality of life, mental health, and stress levels. Our study aims to investigate the level of stress caregivers experience in their everyday life and the way stress affects their quality of life. *Materials and Methods*: We conducted a multicenter, cross-sectional study from 11 November 2023 to 20 March 2024 in healthcare units in Western Greece. All 96 participants were adult caregivers of patients diagnosed with multiple sclerosis (MS). We assessed caregivers’ quality of life and stress levels using the 36-item Short Form Health Survey and Kingston Caregiver Stress Scale, respectively. Non-parametric tests (Spearman’s rho test, Kruskal–Wallis and Mann–Whitney tests) were used to identify possible correlations between the aforementioned scales and between stress levels and caregiver characteristics. *Results*: Caregivers reported high stress, with an average KCSS score of 36.82 ± 0.851. The mean SF-36 physical component summary score (PCS) was 59.59 ± 2.77, whereas the mental component summary score (MCS) was 45.69 ± 2.32. Stress levels in the KCSS were negatively correlated with both PCS and MCS of SF-36 and female gender, education level, monthly income, limits of mobility, and patient’s level of dependence were associated with higher levels of stress. *Conclusions*: Stress was found to be negatively connected with caregivers’ quality of life, affecting both physical and mental health. Female caregivers, caregivers with a primary education level and a low monthly income, and caregivers of patients with serious illnesses who rely primarily on daily help were the most affected.

## 1. Introduction

Multiple sclerosis (MS) is a neurodegenerative and autoimmune disease affecting 2.9 million people worldwide (35.9 per 100.000 population), with increasing prevalence in every world region since 2013 [1,2]. This condition typically develops in the third or fourth decade of life. MS is equally prevalent among preteen children, both boys and girls. During adolescence, the curves begin to diverge, with girls experiencing a higher prevalence than boys. This tendency continues until approximately the end of the sixth decade of life, when the gender ratio is 2:1 in favor of women [3]. MS is defined by periods of neurological symptoms that are frequently followed by neurological dysfunctions that lead to increased disability over 30–40 years [4,5]. It is one of the most common causes of neurological disability in young adults [6]. While the age-standardized mortality rate for multiple sclerosis decreased by 11.5 percent globally in 2016 and new treatments are continuously being developed to manage the disease’s symptoms, the global disability-adjusted life years (DALYs) for multiple sclerosis in 2016 were 1,151,478 (95% UI 968,605 to 1,345,776), indicating a non-significant decrease of 4.2% (95% UI −16.4 to 0.8) from 1990 [3]. 

Patients with multiple sclerosis (MS) frequently require daily assistance due to the disease’s diverse and progressive nature, which presents in a variety of physical and cognitive deficits [7]. Motor dysfunction, which includes weakness, spasticity, and ataxia, can have a substantial impact on daily activities like mobility, self-care, and ambulation. Seventeen years after disease onset, more than 10% of patients may reach a score ≥6 on the EDSS (EDSS: Expanded Disability Status Scale), and more than 18% will evolve from relapsing MS to secondary progressive MS (SPMS) [8]. Increased disability leads in terms of challenges in daily living as patients may be unable to deal with their work, resulting in unemployment [9]. Furthermore, sensory disorders including numbness, tingling, and neuropathic pain are observed in 80% of patients with MS [10]. Sensory abnormalities and pain can impair a patient’s ability to perceive and respond to their surroundings [11]. Cognitive impairment concerns more than 33% of patients with MS, which includes memory, attention, and executive function deficiencies, can make decision-making and independent functioning difficult [12,13]. Moreover, fatigue, a defining symptom of MS, can increase functional impairments, necessitating rest and recuperation throughout the day [4].

To summarize, the disease’s unpredictable nature means that patients frequently require ongoing assistance with daily chores, medical appointments, and emotional support. Caregivers take on this role, offering physical, emotional, and logistical care that can considerably improve the patient’s quality of life [5]. Additionally, as the individual’s disability rises, the role of the caregiver frequently gets more demanding and stressful. The strict nature of caregiving can result in emotional tiredness, physical strain, and social isolation [14]. Caregivers may suffer increased stress and anxiety as they navigate the uncertainties and complexities of MS management, often feeling overwhelmed by the need to constantly adapt to changing conditions. Witnessing the disease’s progression and impact on the life of their close one can cause feelings of loss, frustration, and helplessness [15]. Furthermore, the constant demands of caregiving might lead to disregarding one’s own needs, resulting in damaged physical and mental health. Consequently, caregivers are at heightened risk of experiencing burnout, chronic stress, and a spectrum of physical ailments, including cardiovascular diseases and musculoskeletal injuries, due to the sustained physical and emotional labor involved [16]. Moreover, the psychological toll, manifesting as anxiety, depression, and social isolation, exacerbates the strain on caregivers, culminating in a significant decline in their overall quality of life and health outcomes [17]. For all these reasons, it is critical to consider caregivers’ physical and mental health, and their overall quality of life, shifting from a patient-oriented strategy to one that includes both patients and caregivers, because caregivers are both “hidden patients” and “cotherapists”, and their well-being is fundamental to the patients’ well-being [18]. 

Our study seeks to measure the quality of life and the levels of everyday stress in caregivers of patients with MS, to determine further possible correlations between those two, and to identify potential relationships between levels of stress and characteristics of caregivers (age, gender, duration of caregiving, income, education, place of residency) and the severity of the patient disability. Our main purpose is to emphasize that the diagnosis of multiple sclerosis, even in patients with mild or subclinical symptoms, causes considerable stress for those who are close to them and take on the role of caregiver. We aim to underline the importance of a holistic approach to health, moving away from the patient–physician polarity based solely on pharmaceutical therapy and implementing a therapeutic plan that includes both the patient and their entire intimate environment, including the caregiver. Our work contributes to the literature on caregiver burden, as there are few studies in our country and around the world, despite growing interest in a more holistic approach to health.

## 2. Materials and Methods

This multicenter cross-sectional study took place from 11 November 2023 to 20 March 2024. We used a convenience sampling method, and all 96 participants were adults attending urban-type health centers or outpatients’ clinics in the city of Patras, in the Western Greece region. The Ethics Committee of the University of Patras approved the study protocol (Number ID 15433). The study was performed according to the Declaration of Helsinki.

Participants were caregivers of patients diagnosed with MS, attending the three urban-type health centers or hospital outpatients’ clinics in the city of Patras, in the Western Greece region. For the caregivers, inclusion criteria were as follows: (1) the patient they helped should have a definite diagnosis of MS, (2) an age greater than 18 years old, (3) helping the patient and being responsible for his/her everyday care and well-being, (4) knowledge of writing and reading, and the citizen used the primary health care unit themselves at the given time. Exclusion criteria were being (1) caregivers of hospitalized patients, (2) caregivers of patients who had not yet received a definite diagnosis of MS, (3) citizens who provided temporary and non-permanent care to patients with MS, or citizens who voluntarily participated in patient support groups.

Participation in the survey was voluntary and anonymous. Participants, after being informed by the researcher, provided their written consent for participation in the study and analysis and publication of the data that would result from it. Then, they were asked to fill in individual questionnaires. 

There were three parts to the questionnaire. The first part consisted of demographics and short medical history questions [patient’s and caregiver’s gender, patient’s and caregiver’s age, educational level, marital status, monthly income, place of residence, the existence of a chronic disease in the caregiver’s medical history, patient’s limits of mobility, patient’s dependence level, the length of time care was provided, and the type of care provided (Table 1)].

The caregivers’ quality of life was the subject of the second part of the questionnaire. We used the validated Greek version of the 36-item Short Form Health Survey (SF-36) [19]. The SF-36 measures physical and mental health in the following eight dimensions: vitality, physical functioning, body pain, general health perceptions, physical role functioning, emotional role functioning, social role functioning, and mental health. These eight dimensions are scaled so that the total score of the SF-36 ranges from 0 to 100, with lower scores meaning greater disability [20]. 

In the third part, stress was assessed with the Greek version of the Kingston Caregiver Stress Scale (KCSS) [21], which consists of ten questions dealing with caregiving, family, and financial status (seven, two, and one question, respectively). Each answer is scored from 1 (no stress) to 5 (extreme stress); the total stress score for caregivers is computed by adding together all answers. Three groups emerge: mild stress levels range from 10 (the lowest) to 14, moderate stress levels range from 15 to 23, and severe stress levels range from 24 to 50. Pitsikali et al. [21] reported that the KCSS is especially suitable for assessing unpaid caregivers, usually the partner or other relatives of the patient, and has satisfactory psychometric properties.

In order to decide which was the most appropriate statistical test, we first checked to determine if the KCSS index axes followed a normal distribution. As the KCSS index proved to not have a normal distribution, non-parametric tests were used. Frequency tables were used for a descriptive analysis of the data; the non-parametric Spearman’s rho test was used to investigate the relationship between the stress levels obtained from the KCSS and the physical (PCS) and mental (MCS) component summary scores of SF-36; and the non-parametric Kruskal–Wallis (Kruskal–Wallis test) and Mann–Whitney (Mann–Whitney test) tests were used to investigate the KCSS with the special characteristics of the population under consideration. Double entry tables and boxplots were used to present the findings.

The data were analyzed using the statistical tool IBM SPSS Statistics version 28.0.1.1 (Statistical tool for Social Sciences), and the results were applied to make inferences and correlate various factors.

## 3. Results

Ninety-six caregivers of patients diagnosed with MS were enrolled in the study. Their baseline characteristics are presented in Table 1. The majority of our caregivers were either the patient’s husband or wife, or a first-degree relative (parent, sibling, or child).

Caregivers experienced significant stress, with an average KCSS score of 36.82 ± 0.851 (Table 2). Accordingly, the mean value of the SF-36 physical component summary score (PCS) was 59.59 ± 2.77, while the mental component summary score (MCS) was 45.69 ± 2.32 (Table 2).

Stress levels in the KCSS were negatively correlated with both PCS and MCS of SF-36 (Table 3). More specifically, increased levels of stress in the KCSS were associated with a lower score in the PCS (Correlation Coefficient −0.405, *p*-value < 0.001) and in the MCS (Correlation Coefficient −0.401, *p*-value < 0.001).

In the univariable logistic analysis of the associations of nonordinal baseline characteristics and stress level in the KCSS, the caregiver’s age, educational level, monthly income, mobility limitation, and the patient’s dependence level were associated with a higher level of stress in the KCSS (Table 4).

**Figure 1:**
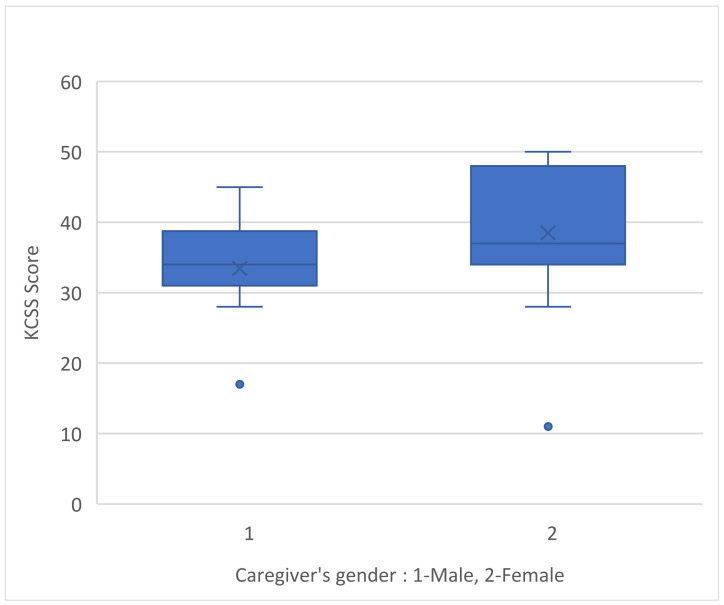
KCSS score and caregiver’s gender.

Between the male and female caregivers, female caregivers reported higher levels of stress (*p*-value 0.007) (Figure 1).

**Figure 2:**
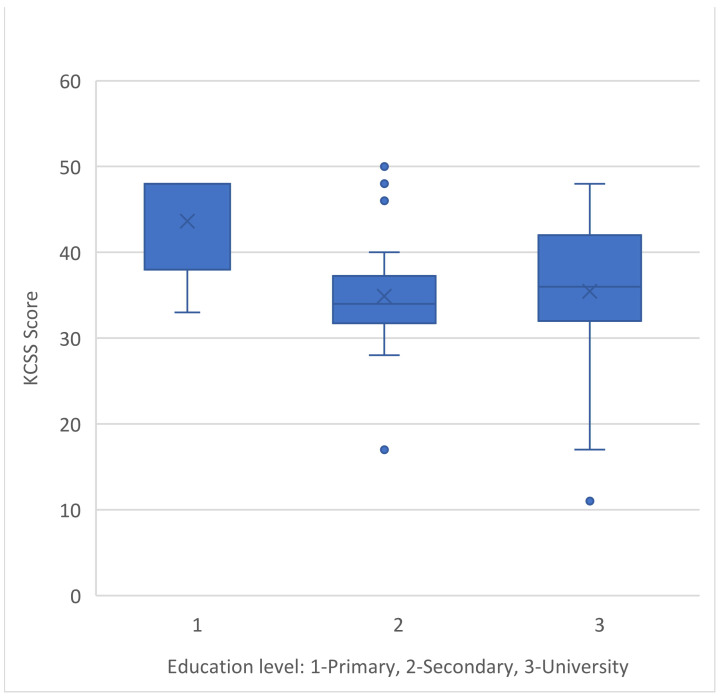
KCSS score and caregiver’s education levels.

Caregivers with a primary education level reported greater levels of stress in the KCSS compared to those with higher levels of education (Kruskal–Wallis H = 20,574/df = 2/*p*-value < 0.001) (Figure 2).

**Figure 3:**
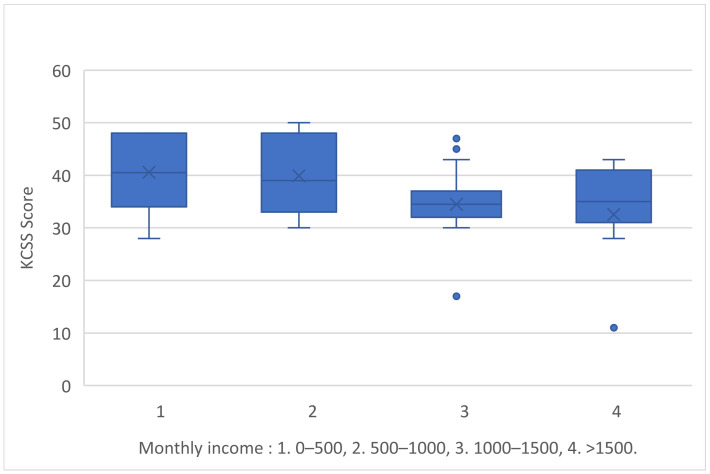
KCSS score and caregiver’s monthly income.

Compared to the caregiver’s monthly income, the two groups with less income had higher levels of stress than the other two groups with higher incomes (Kruskal–Wallis H = 11,715/df = 3/*p*-value = 0.008) (Figure 3).

**Figure 4:**
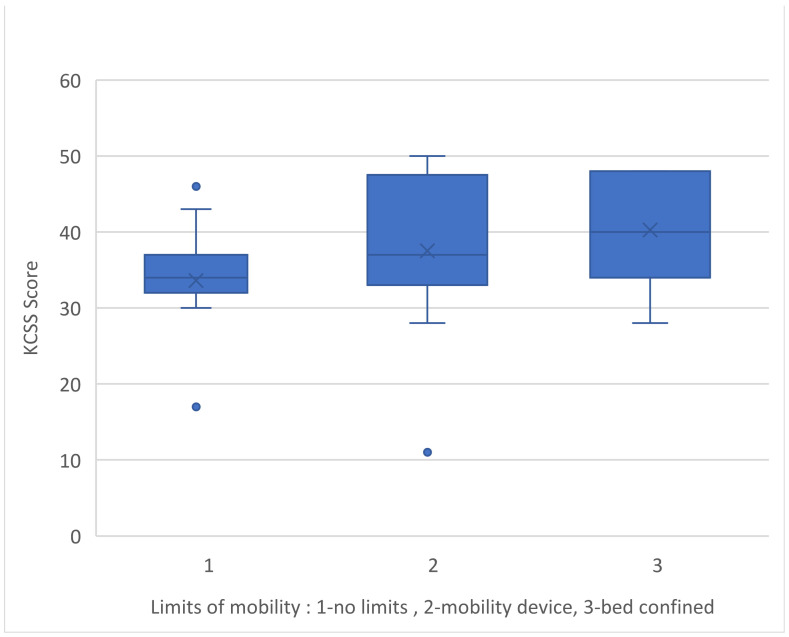
KCSS and patient’s limits of mobility.

Finally, there was a correlation between stress levels in the KCSS and mobility, as well as the patient’s degree of dependence. Limits of mobility and degree of dependence are among the features associated with disease severity. More precisely, caregivers of patients who were confined to beds reported higher levels of stress compared to the other groups (Kruskal–Wallis H = 10,492/df = 2/*p*-value = 0.005) (Figure 4).

**Figure 5:**
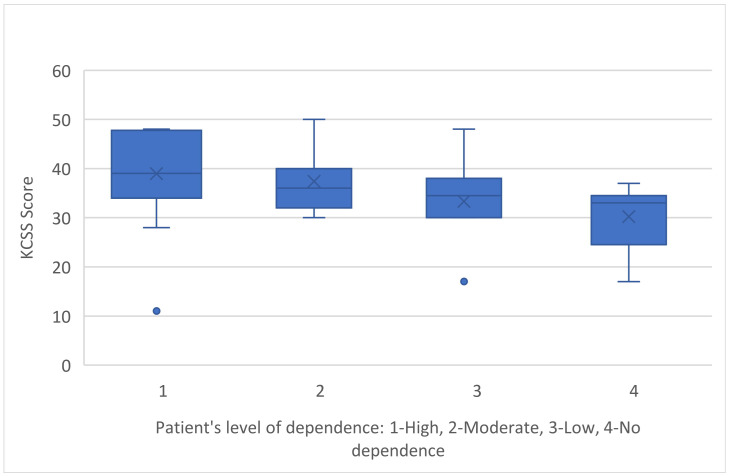
KCSS and patient’s level of dependence.

Furthermore, as the degree of dependence of the person with sclerosis on the caregiver increased, so did the stress levels, with caregivers who cared for individuals with a high or moderate degree of dependence showing a statistically significant difference from the other two groups, with little or no dependency (Kruskal–Wallis H = 12,896/df = 3/*p*-value = 0.005) (Figure 5).

## 4. Discussion

The outcomes of our study demonstrate the significant degree of stress experienced by caregivers of MS patients, which are consistent with prior studies [12,15,22,23,24,25]. Caregivers suffer from substantial levels of stress, as shown by their mean KCSS score of 36.82 ± 0.851 (KCSS subjective level of stress: 10–14 mild, 15–23 moderate, 24–50 severe [21]). In addition, caregivers reported a diminished quality of life (PCS and MCS ratings on the SF-36: 50.8 ± 9.2 and 42.8 ± 12.2, respectively). Our study is consistent with the literature [26] since the results suggested that the highest levels of stress were experienced by female caregivers, as well as caregivers with a low level of education and a low monthly income.

Caregiver’s stress was correlated with both PCS and MCS of SF-36 underlining something that is in line with plain sense: excessive levels of stress affect all aspects of the quality of life of caregivers, both physically and mentally. Caregivers who have to contend with the unpredictable course of the disease endure a full fragmentation of their level of fitness, self-image, satisfaction with family life, work, the economic position, interaction with others, social support, and overall life [27]. Despite their unshakable devotion and dedication, caregivers may experience feelings of shame, inadequacy, and resentment while attempting to balance their personal needs with the duties of caregiving [22].

The current study observed that a caregiver’s emotional burden was associated with the caregiver gender, with female respondents reporting higher levels of stress on the KCSS (*p*-value = 0.007). Although there are no similar studies in the Greek area that confirm the above negative correlation [15,22], one interesting interpretation for the above result, based on a sociological approach, is that women’s complex and demanding roles in the family often cause stress and emotional burden [28]. Women’s emotional health can be severely impacted by gender stereotypes that define their role by emphasizing the aspect of care that they “must” effectively offer in addition to all of their other responsibilities [26,29].

The caregivers’ educational level additionally had a significant role in our findings, as we identified that caregivers with only a primary education level experienced higher levels of stress (KCSS) than caregivers with two higher education levels. This outcome may be explained by the fact that those with lower levels of education probably have fewer opportunities to pursue careers, and as a result, they prioritize their job as caregivers over any other roles they may play in life [15]. Another reason could be that they suffer a greater asymmetry of knowledge in the information provided by the attending physician about the disease and its treatment, have trouble following the course of the condition, and therefore experience more uncertainty and fluctuation [30,31]. Employment appears to improve the well-being of caregivers, which is linked to people’s ability to find purpose and meaning in life. Furthermore, according to Bassi M. et al. [32], working life functions as a “protective factor” against burnout among caregivers.

Caregivers with lower monthly income (EUR 0–500 and EUR 500–1000) expressed more stress than those with greater income. It is evident that suffering from a chronic disease causes a psychological and physical strain on the caregiver, as well as a financial burden [33]. Expensive medicine, physical and occupational therapy rehabilitation, and other needs of persons with multiple sclerosis are the primary concerns, generating stress and anxiety for patients and caregivers [34].

Disease progression has been linked to increased stress levels. More precisely, caregivers of patients with physical disability experienced higher levels of stress, as did caregivers of patients who required constant assistance to function on a daily basis. Regarding this association, Garre-Olmo J. et al. [35] state that the degree of dependency and mobility limits are directly correlated with the severity of the condition, which increases the stress that caretakers endure.

Addressing the unique challenges faced by caregivers of multiple sclerosis (MS) patients necessitates the implementation of specific interventions and the provision of targeted resources designed to mitigate stress and enhance overall well-being. Educational programs and self-management programs play an important role in providing caregivers with complete knowledge about disease progression, symptom management, and effective caregiving practices, allowing them to undertake everyday caring chores with better confidence and competence [36,37]. Self-help books, weekly telephone calls, psychoeducational training, seems to improve caregivers’ understanding of the disease and help them cope with everyday challenges [37]. Furthermore, involvement in support groups designed expressly for MS caregivers provides an important platform for emotional support and the exchange of practical information, promoting a sense of community and minimizing feelings of loneliness [38]. Other forms of support, such as Acceptance and Commitment Therapy (ACT) could also provide support and the acceptability and feasibility of this intervention should be studied [39]. Respite care services are another crucial intervention that provides caregivers with short reprieve from caring obligations while also allowing them to recover physically and mentally. These services, which range from in-home respite care to short-term residential care facilities, provide flexibility to meet a variety of needs.

Clinically, these interventions can lead to better management of caregiver stress, a lower incidence of burnout, and improved mental health, all of which improve the quality of care offered to MS patients [37]. These findings highlight the need for healthcare systems and policymakers to allocate funds to develop and sustain caregiver support programs. Integrating such personalized interventions into standard care methods can result in a more holistic approach to chronic illness management, addressing the needs of both patients and caregivers. This dual-focus approach has the potential to lower healthcare costs by reducing the number of hospital admissions and delaying the need for long-term institutional care for patients, emphasizing the necessity for complete caregiver support in chronic disease management frameworks.

Despite our study’s strengths, which include its design with the use of validated tools to measure caregivers’ stress and quality of life and the enrollment of caregivers of patients attending not only hospital outpatient’s clinics but also other healthcare units, such as urban-type health centers and primary healthcare units, some limitations must be addressed. First, because our sample was small and representative of an urban population in Western Greece, the findings may not be relevant to other groups. Second, limitations arise from the sampling method used (convenience sampling). Beyond that, the study’s duration (November–March) was not annual, raising the possibility that the results were influenced by the unique characteristics of the time period. Finally, another limitation is the absence of data on disease phenotype and the lack of PDDS/EDSS as a measure of the pwMS disability. However, when designing the study, we did not intend to investigate the unique characteristics of subgroups of caregivers but rather to see if the diagnosis of multiple sclerosis alone, even in patients with mild symptoms and no motor disability, could cause significant levels of stress in caregivers. This was also an important conclusion that our study contributed to the literature.

## 5. Conclusions

Our study, one of the few designed and carried out in the Greek area, is consistent with the existing literature since it found that anxiety was negatively correlated with caregivers’ physical health status and mental health status. Additionally, caregiver strain was positively correlated with the severity of illness, patient’s dependence level, education, gender, and monthly income of the caregiver. Considering that most caregivers come from family backgrounds and are unpaid, and that high levels of stress can put the caregiver’s health at significant risk and subsequently burden the health of the person with MS from the resulting lack of care, it is necessary to offer caregivers the necessary support, financial, mental, and social, so as to reduce the degree of the caregivers’ burden.

## Figures and Tables

**Table 1:** Baseline characteristics of the caregivers.

		*n*	%
Caregiver’s gender	Male	32	33.3%
	Female	64	66.7%
Patient’s gender	Male	45	46.9%
	Female	51	53.1%
Caregiver’s age	18–44	35	36.5%
	45–65	53	55.2%
	>65	8	8.3%
Patient’s age	18–44	50	52.1%
	45–65	39	40.6%
	>65	7	7.3%
Marital status	Unmarried	12	12.5%
	Married	65	67.7%
	Divorced	12	12.5%
	Single parent	2	2.1%
	Widowed	5	5.2%
Education level	Primary	19	19.8%
	Secondary	42	43.8%
	University	35	36.5%
Monthly income	<EUR 500	14	14.6%
	EUR 500–1000	31	32.3%
	EUR 1000–1500	36	37.5%
	>EUR 1500	15	15.6%
Place of residence	Urban	63	65.6%
	Semi-urban	21	21.9%
	Rural	12	12.5%
Limits of mobility	No limits	36	37.5%
	Mobility device	33	34.4%
	Bed-confined	27	28.1%
Degree of consanguinity	1st degree	44	45.8%
	Other degree	45	46.9%
	No degree	7	7.3%
Duration of the caregiving	<1 month	2	2.1%
	1–6 months	9	9.4%
	6–12 months	12	12.5%
	1–2 years	6	6.3%
	2–4 years	17	17.7%
	>5 years	50	52.1%
Type of care provided	Personal care	5	5.2%
	Household	3	3.1%
	Emotional–psychological support	15	15.6%
	Financial	7	7.3%
	Other	3	3.1%
	All of the above	63	65.6%
Patient’s level of dependence	High	48	50.0%
	Moderate	23	24.0%
	Low	16	16.7%
	No dependence	9	9.4%
Chronic disease in caregiver’s medical history	Yes	34	35.4%
	No	62	64.6%

**Table 2:** Descriptive statistics of KCSS, SF-36 PCS, and SF-36 MCS of the study population.

		Statistic	Std. Error
KCSS Score	Mean	36.82	0.851
	Median	36.00	
	Std. Deviation	8.334	
SF36_PCS	Mean	59.59	2.775
	Median	64.07	
	Std. Deviation	27.189	
SF36_MCS	Mean	45.69	2.325
	Median	47.02	
	Std. Deviation	22.782	

**Table 3:** Spearman’s rho test for the strength of the association between KCSS, SF-36 PCS, and SF-36 MCS.

			KCSS Score	SF36_PCS	SF36_MCS
Spearman’s rho	KCSS Score	Correlation Coefficient	1000	−0.405 **	−0.401 **
		Sig. (2-tailed)	.	<0.001	<0.001
		N	96	96	96

**: Correlation is significant at the 0.01 level (2-tailed).

**Table 4:** Univariable analysis of the associations of nonordinal baseline characteristics and KCSS.

KCSS		**Mann–Whitney U**	**Wilcoxon W**	Z	***p*-Value**
	Caregiver’s gender	680,500	1,208,500	−2.679	0.007
		Kruskal–Wallis H		df	*p*-value
	Education level	20,574		2	<0.001
	Monthly income	11,715		3	0.008
	Limits of mobility	10,492		2	0.005
	Patient’s dependence level	12,896		3	0.005

## Data Availability

The data that support the findings of this study are available from the corresponding author upon reasonable request.
